# Innovative Electrospun Nanofiber Mats Based on Polylactic Acid Composited with Silver Nanoparticles for Medical Applications

**DOI:** 10.3390/polym16030409

**Published:** 2024-02-01

**Authors:** Tongsai Jamnongkan, Kawisara Sirichaicharoenkol, Vanida Kongsomboon, Janitsata Srinuan, Natee Srisawat, Autchara Pangon, Rattanaphol Mongkholrattanasit, Achiraya Tammasakchai, Chih-Feng Huang

**Affiliations:** 1 Department of Fundamental Science and Physical Education, Faculty of Science at Sriracha, Kasetsart University, Chonburi 20230, Thailand; kawisara.s@live.ku.th (K.S.); vanida.k@live.ku.th (V.K.); janitsata.s@live.ku.th (J.S.); 2 Department of Textile Engineering, Faculty of Engineering, Rajamangala University of Technology Thanyaburi, Pathumthani 12110, Thailand; natee.s@en.rmutt.ac.th; 3 Nano Functional Fiber Research Team, National Nanotechnology Center, National Science and Technology Development Agency, Pathumthani 12120, Thailand; autchara@nanotec.or.th; 4 Faculty of Industrial Textiles and Fashion Design, Rajamangala University of Technology Phra Nakhon, Bangkok 10110, Thailand; rattanaphol.m@rmutp.ac.th; 5 Department of Anatomy, Faculty of Medical Science, Naresuan University, Phitsanulok 65000, Thailand; achirayata@nu.ac.th; 6 Department of Chemical Engineering, i-Center for Advanced Science and Technology (iCAST), National Chung Hsing University, Taichung 40227, Taiwan

**Keywords:** nanofibers, biocomposites, poly(lactic acid), silver nanoparticles, antibacterial properties

## Abstract

Nanofibers are some of the most attractive materials that can modify functionalities for developing new kinds of specific applications and are mainly used as a biomedical material. Herein, we designed and prepared antibacterial nonwoven fiber mats of PLA and PLA composited with Ag nanoparticles by electrospinning. The effects of varying filler contents on their chemical, surface morphology, thermal, water absorbency, and antibacterial properties were investigated using FTIR, SEM/EDS, DSC, swelling ratio, and qualitative and quantitative antibacterial tests. FTIR and EDS spectra indicated that Ag nanoparticles were incorporated in the PLA without chemical bonding. SEM revealed that the average diameter of the PLA nanofibers containing the Ag nanoparticles was more significant than those without those particles. In addition, fiber diameters are proportional to the amount of Ag nanoparticle contents. DSC indicated that the Ag nanoparticles can be incorporated within the PLA matrix without strongly affecting their thermal properties. Moreover, the crystallinity of the composite nonwoven fiber mats was higher than those of fiber mats in the neat PLA. However, TGA revealed that the loaded Ag can improve the thermal stability of the PLA electrospun fiber mats. Accordingly, the antibacterial activities revealed that all the composite nanofiber mats exhibited excellent resistance against *S. aureus* and *E. coli* bacterial strains. In addition, in the cell toxicity study, all produced hybrids of nonwoven fiber mats induced a reduction in cell viability for the L929 fibroblast cells. Our results suggest that the designed and prepared nonwoven fiber mats may have good potential for use in the biomedical field, particularly in wound dressing applications.

## 1. Introduction

Nowadays, nanotechnology is an exciting research topic for researchers in academia and industrial fields due to it forming matter at the nano range with more enhanced features compared to its bulk counterparts. Recently, nanofiber is one of the nanotechnologies that has widely attracted attention from several researchers due to its many specific properties such as it being lightweight and having a large surface-to-volume ratio and high porosity [1,2,3,4]. These appropriate nanofiber properties are especially suitable for use as a material for several applications, i.e., filters [5] and catalysts [6]. It is well known that nanofibers can be fabricated using various techniques. Among those techniques, electrospinning is a versatile, widely studied, and commonly employed technique used to produce nanofibers with unique properties, as we mentioned, such as high surface area-to-volume ratio, high porosity, tunable features, and the cost-effective process of manufacturing nanofibers [7,8,9,10]. Therefore, electrospinning is the most efficient way to prepare nanofibers with synergistic characteristics for new applications by blending multiple polymers with individual functionalities in the solution phase. The morphology of electrospun fiber mats depended on the processing and polymer solution spinning parameters like the applied voltage, polymer flow rate, solution concentration, molecular weight of the polymer, relative humidity, diameter of the needle, and tip-to-collector distance [11,12,13,14]. These nanofibers find applications in various fields, including tissue engineering [15,16,17], filtration [18,19,20], energy storage [21,22], adsorbents [23,24,25], and drug delivery [26,27,28,29]. In recent years, electrospinning has also emerged as a promising method for producing antibacterial nanofibers due to their potential in combating microbial infections and their application in wound healing [30,31,32,33]. This technique offers numerous enticing possibilities, one of which involves the inclusion of drugs, functional fillers, or bioactive molecules within the core of electrospun fibers [34].

Over the past few decades, using polymeric materials as nonwoven fiber mats made by electrospinning processes has been a popular approach in medical applications. For example, Ullah et al. [35] prepared an ultrafine bioactive among oil-loaded electrospun cellulose acetate nanofibers for wound dressing applications. They also reported that these fibrous materials exhibited in vitro biocompatibility, cell viability, and good antibacterial and antioxidant properties. They suggested that these nonwoven electrospun fiber mats can be used to heal wound infections. Hashemikia et al. [36] also prepared electrospun fiber mats of chitosan/polyethylene oxide/silica nanofibers composited with ciprofloxacin for applying to wound healing. They also investigated in vitro and in vivo drug delivery. They mentioned that the silica particle played a crucial role in collagen creation, accelerating wound healing. Furthermore, the essential organic compounds from natural products have attracted attention as a functional drug for application in the electrospun fiber mat. Santos and coworkers [37] fabricated the electrospun fiber mats of cellulose acetate composited with annatto, its natural dye exhibiting anti-inflammatory, antioxidant, and antimicrobial properties, for wound dressing. These functionalized materials exhibit the results of inflammatory process and biocompatibility. However, we also found that several types of materials are used in electrospinning to produce electrospun nanofibers, i.e., poly(vinyl alcohol) [38], poly(ethylene glycol) [39], polyamide [40], poly(vinylidene fluoride) [41], polycaprolactone [42], and chitosan [43].

As a safe biopolymer, polylactic acid (PLA) is one of the candidate materials for fabricating electrospun fiber mats due to its several advantageous properties, such as non-toxicity, good transparency, biodegradability, biocompatibility, and good thermal and mechanical performance [44,45,46,47,48,49]. Consequently, many studies have already demonstrated the application of electrospun PLA as a direct material for specific applications in various fields. However, it is well known that neat PLA exhibits brittle and low flexibility properties. Therefore, blending, or composites with different materials, is beneficial for improving their properties. Presently, the functionalities of biocomposite nanofibers (fabricated from biodegradable matrices reinforced with fillers) are becoming of utmost interest as an environmentally friendly materials with potentially specific applications, particularly in the medical fields. The easiest way to make functional electrospun fiber mats is to add the functionality materials into a polymer solution prior to the spinning process. Recently, producing PLA-based nonwoven fiber mats through the electrospinning technique has received growing attention. Several previous studies have stated that functionalized electrospun fiber mats with antimicrobial properties were prepared from PLA composited with metal-based nanoparticles such as silver (Ag) [50,51,52], zinc oxide (ZnO) [53], and titanium dioxide (TiO_2_) [54,55]. Hence, it is not surprising that PLA has been widely used to produce nanofibers and help inorganic materials fabricate nanofibers’ functionalities for various applications. Regarding the above previous reports and observations, there are only directly applied nanoparticles, without any precursor of nanoparticles, into the polymer solution. This results in their nanoparticle’s trend to aggregation or agglomeration within the medium of polymer solution, which caused their physical properties, including the efficacy of antibacterial activity as well. To resolve these problems here, we intend to use a single-step method for the synthetic preparation of colloidal Ag–NPs during the spinning process, with the present system finding applications in the biomedical field.

Therefore, the present work is an extensive study of the implementation of the fabrication of antimicrobial nonwoven fiber mats based on PLA loaded with Ag nanoparticles by electrospinning techniques. Here, Ag nanoparticles were prepared in a single step by reducing silver nitrate (AgNO_3_), used as a precursor, while preparing the PLA solution for electrospinning. It is well known that the antibacterial efficacy of Ag nanoparticles also depends on their contents, which increases their antibacterial properties due to the higher opportunities for penetration of the bacteria cells. Therefore, the effects of the Ag concentrations within the polymer suspension on the morphology and physical properties of the obtained nanofibers were investigated. Additionally, the chemical interaction, morphology surface, thermal properties, water absorbency, and antibacterial properties were examined by using Fourier transform infrared spectroscopy (FTIR), scanning electron microscope (SEM), differential scanning calorimetry (DSC), thermal gravimetric analysis (TGA), swelling ratio, as well as qualitative and quantitative antibacterial tests, respectively.

## 2. Materials and Methods

### 2.1. Materials

Commercial-grade Poly (lactic acid) (PLA) was obtained from NatureWorks LLC (Plymouth, MN, USA) with the trade name of Ingeo 3100 HP (*Mw* = 140,000 g/mol). Silver nitrate was purchased from LOBA Chemie Co., Ltd., Mumbai, India. Dichloromethane and dimethylformamide were purchased from Sigma Aldrich-Merck Co., Ltd., Oberbayern, Germany. Other chemical agents were analytical-grade purity and used as received without further purification.

### 2.2. Electrospinning Process

Here, the biocomposite nanofibers were fabricated by using the electrospinning process as follows: First, PLA powders were dissolved in a cosolvent of dimethylformamide and dichloromethane at the ratio of 50:50 at a concentration of 10 wt% at 80 °C for 3 h. Approximate amounts of silver nitrate (for the desired concentrations of 1, 3, and 5 wt%) were added into the 10 wt% PLA solution and continuously stirred for 12 h to obtain homogeneous solutions. Second, the polymer suspensions were treated with ultrasonication to destroy small air bubbles in the solution. Third, the suspension was loaded into a 10-mL plastic syringe. A metal needle was fitted to a syringe and connected to a high-voltage generator. A rotating metal wrapped with aluminum foil and connected to the ground was used as the collector for the composite nanofibers. The polymer suspension was pumped through a syringe controller at a flow rate of 0.05 mL/h during electrospinning. The syringe used in this experiment had a capillary tip diameter of 21 gauge and contained an attached copper wire used as the positive electrode. A voltage of 10 kV was applied to the spinning solution, and a dense web of fibers was collected on the aluminum foil. The nanofibers were collected on a metal collector wrapped with aluminum foil and kept at a fixed distance of 10 cm away from the needle tip of the spinneret. All conditions were performed at room temperature, approximately 25 ± 5 °C, and the relative humidity at 50± 2%. The obtained nonwoven fiber mats were dried initially at 60 °C for 8 h prior to further characterization. In this paper, to be convenient for discussing the experimental results, the samples are referred to as PLA/Ag-1, PLA/Ag-3, and PLA/Ag-5, which correspond to a 10 wt% PLA solution in which silver nitrate is suspended at concentrations of 1, 3, and 5 wt%, respectively.

### 2.3. Scanning Electron Microscope

The surface morphology of all fabricated nonwoven fiber mats was examined through SEM (Model: Quanta 450, FEI, Hillsboro, OR, USA). Before analysis, all samples were cut into small pieces (1 × 1 cm^2^) and sputter coated with a thin layer of gold. All images were captured and operated using secondary electrons and 15 kV of accelerating voltage after the sputter coating of specimens with a gold nanolayer. Moreover, the nanofiber’s dimensions, average fiber diameter and distribution, were investigated via image analysis using ImageJ software (ImageJ, version 1.45, USA) from at least 100 measurements per sample.

### 2.4. Fourier Transform Infrared Spectroscopy

FTIR was performed to examine the chemical interaction between silver nitrate molecules and PLA molecular chains. An FTIR spectrometer (Model: Invenio, Bruker, Billerica, MA, USA) in ATR mode equipped with a diamond crystal was used for investigation. All the spectra were recorded in transmittance mode with 4 cm^−1^ resolution in the wavenumber range of 4000 cm^−1^–400 cm^−1^ under ambient conditions.

### 2.5. Differential Scanning Calorimetry (DSC) Analysis

Thermal properties of nonwoven fiber mats based on PLA were characterized using a DSC technique (Model: DSC 200 F3, Netzsch, Barrie, ON, Canada). All the samples were in a sealed aluminum pan and each piece used was approximately 5.0 mg. They were sequentially heated from 30 to 200 °C, cooled to 25 °C, and then reheated to 200 °C at the rate of 10 °C/min under a nitrogen flow of 50 mL/min. Then, the degree of crystallinity of the obtained nonwoven fiber mats ($\chi_{c}$) was calculated by Equation (1) [56]

(1) $$\chi_{c}\left[\%\right]=\frac{\Delta H_{m}}{w\times \Delta H_{m}^{o}}\times 100$$

where ${∆H}_{m}^{o}$ is the melting enthalpy for 100% crystalline PLA (93.0 J/g) [57]; ${∆H}_{m}$ is the melting enthalpy for the nonwoven fiber mats; and w is the mass fraction of PLA in the fiber mats.

### 2.6. Thermal Gravimetric Analysis (TGA)

Thermal stability of the fibers and the composite fibers was performed through the thermogravimetric analyzer (TGA) using a TGA instrument (Model: TGA 7, Perkin-Elmer, Waltham, MA, USA). Here, 5.0 mg of each sample was weighed on the analytical balance and placed in the cell of the TGA instrument equipment. The parameters were set in a nitrogen atmosphere of 20 mL/min, and the analysis was performed in a temperature range of 30 °C to 800 °C at a heating rate of 10 °C/min.

### 2.7. Water Absorbency

The swelling ratio of the nonwoven fiber mats based on PLA was determined from the relationship between the swollen and dry weight of the sample. The procedure for testing has been previously reported as follows [58]: First, the sample of PLA nonwoven fiber mat was weighed to obtain the initial dry weight (W_d_) and dimensions. The nonwoven fiber mat sample was then placed in a beaker containing deionized water and was removed after 5 min. Then, the wet sample was rapidly dried with filter paper to remove the excess water and subsequently weighed to define W_t_. This process continued, with the weight after immersion for selected times. Finally, the water absorbency or swelling ratio (%SR) of each nonwoven fiber mat was also calculated according to Equation (2):

(2) $$\%SR=\frac{W_{s}-W_{d}}{W_{d}}\times 100$$

where W_d_ is the dry weight of the nonwoven fiber mats and W_S_ is the weight of the swollen sample immersed in the de-ionized water at room temperature at the selected times. All the tests were performed in triplicate (*n* = 3) under identical conditions.

### 2.8. Antibacterial Testing

The in vitro antibacterial activities in both qualitative and quantitative tests were investigated in each electrospun fiber sample for evaluation against Gram-negative (*E. coli*) and Gram-positive (*S. aureus*) bacterial strains, using the disk diffusion susceptibility and plate colonies count method described previously [59], respectively. Briefly, the qualitative antibacterial activity of the samples was investigated as follows: The bacteria used were cultured in an LB medium and then incubated at 30 °C for 24 h, which contained approximately 1.5 × 10^6^ CFU/mL (0.5 McFarland). A 10^−2^ dilution of the incubated bacteria was transferred to an LB agar plate. Disc shapes of 5.00 mm diameter were cut from the samples. A nonwoven fiber mat sample of neat PLA was used as a control for comparing the potential antibacterial properties of the other samples. Next, the cut-out discs were placed on the LB agar plates, spread with bacteria, and incubated for 24 h at 37 °C. After the incubation period, the diameters of the bacterial inhibition zone were measured in mm with a transparent ruler. These experiments were performed in triplicate.

In addition, the potential quantitative antibacterial activity in the samples was performed as follows. The bacterial cells, *S. aureus* and *E. coli,* were grown overnight in an LB broth medium at 30 °C to bacteria grown to contain approximately 1.5 × 10^6^ CFU/mL (0.5 McFarland), the same as the disk diffusion susceptibility test. Then, a 10^−2^ dilution of suspensions of grown bacteria were prepared, and the given content of the samples at the ratio of 1:1 by weight was added to the suspensions. The nonwoven fiber mats from the neat PLA were also used as controls. The suspensions were shaken in a rotary shaker (Model XY-80, Taitec, Koshigaya, Saitama, Japan) at the constant speed of 120 rpm for 3 h at 30 °C and were diluted 10-fold repeatedly. One hundred microliters of an aliquot of these cell solutions were seeded onto LB agar using a surface spread plate technique with a glass stick. The plates were incubated at 37 °C for 24 h. The number of viable bacterial colonies (CFU) was counted and then calculated with the initial number of bacterial cells before dilution. The antibacterial efficacy (%R) of the nonwoven fiber mats was calculated according to the following Equation (3):

(3) $$R=\frac{\left(V_{c}-V_{s}\right)}{V_{c}}\times 100$$

where V_c_ is the number of bacterial cells in the presence of the nanofiber from the neat PLA (CFU/mL) and V_s_ is the number of bacterial cells in the presence of those nanofibers from PLA/Ag (CFU/mL). All the experiments were repeated in triplicates, and the results are presented as mean values.

### 2.9. MTT Testing

L-929 cells, a mouse fibroblast cell line (Elabscience, Houston, TX, USA), were cultured in 96-well plates at a density of 7.5 × 10^4^ cells/mL in a completed MEM medium containing 10% FBS and 1% penicillin–streptomycin (*v*/*v*) and incubated for 24 h in a humidified atmosphere of 5% CO_2_ in an incubator at 37 °C. After this time, the completed MEM was replaced by 200 µL of serum-free MEM medium and sterile nanofiber mats made of PLA, PLA/Ag-1, PLA/Ag-3, and PLA/Ag-5 were soaked in each well. After 24 h of cultivation, cells were rinsed twice with PBS pH 7.4 and following MTT cytotoxicity assays were performed. Briefly, MTT solution (100 µL, 5 mg/mL in serum-free medium) was added to the well and incubated at 37 °C for 3 h. The medium was removed and DMSO was then added to completely dissolve the precipitation. The optical density of the solution (OD_sample_) was detected at 570 nm with a microplate reader. Percentage cell viability was calculated using the following Equation (4):

(4) $$cell\;viability\;(\%)=\frac{{OD}_{sample}}{{OD}_{control}}\times 100$$

## 3. Results and Discussion

### 3.1. Scanning Electron Microscope

**Figure 1:**
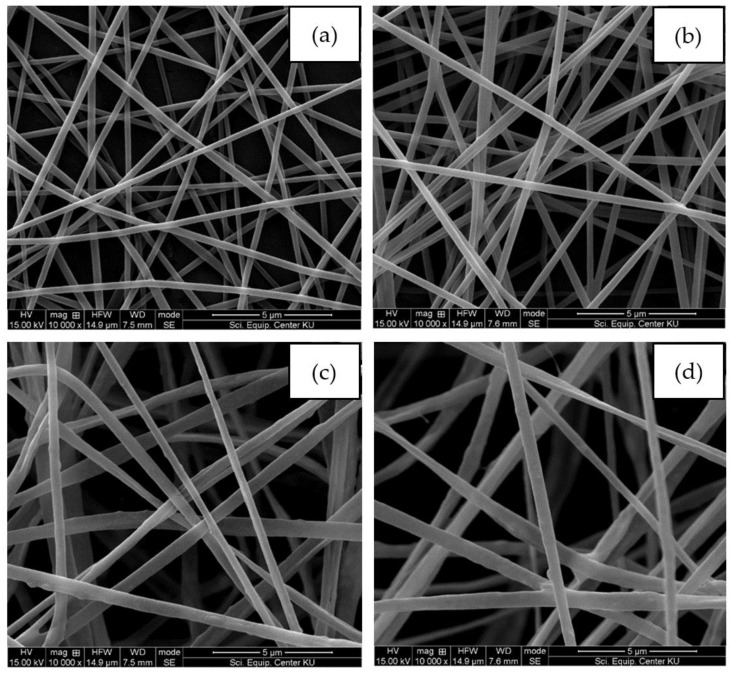
SEM images of the electrospun fiber mats of (**a**) neat PLA, (**b**) PLA/Ag-1, (**c**) PLA/Ag-3, and (**d**) PLA/Ag-5, respectively.

**Figure 2:**
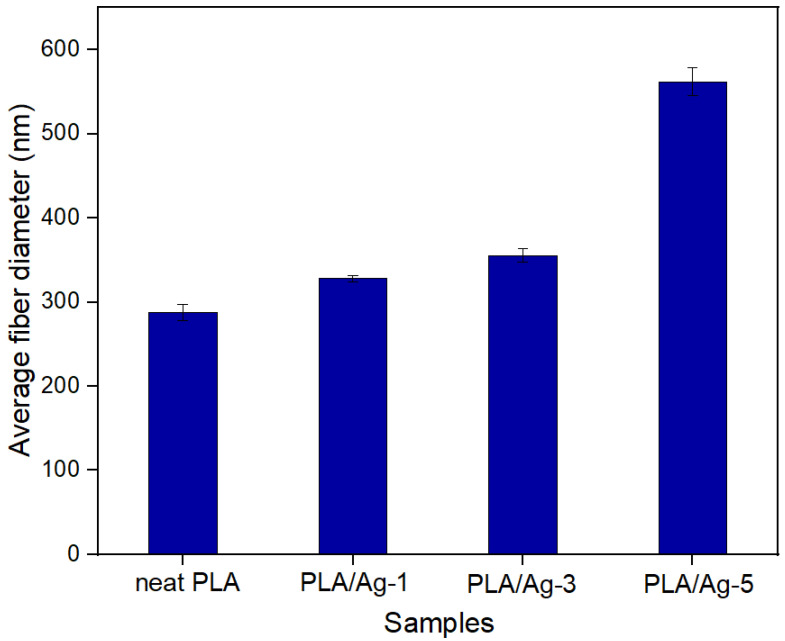
Average fiber diameters of electrospun fiber mats from neat PLA and PLA/Ag at various concentrations.

The morphological surfaces of the nonwoven fiber mats from all samples are shown in Figure 1. We found that all the samples of fabricated electrospun fibers exhibited smooth and uniform fiber diameters without the occurrence of bead defects on the surface. In contrast, from this figure, the size of the electrospun fibers becomes larger with the addition of Ag into PLA matrices. It is well-known that fiber size is one of the main parameters affected by the physical properties of the obtained nonwoven fiber mats. Hence, it is essential to measure this parameter to evaluate the nonwoven fiber mats’ performance. From the experimental results, the average diameters of the nanofibers were approximately 287.80 ± 9.56 nm, 327.96 ± 4.01 nm, 355.33 ± 7.86 nm, and 561.94 ± 16.87 nm for PLA, PLA/Ag-1, PLA/Ag-3, and PLA/Ag-5, respectively. A comparison of the average fiber diameter of electrospun-based PLA structures with different Ag concentrations is illustrated in Figure 2. Interestingly, these results suggest that the addition of Ag increased the average diameter of the nanofibers. In addition, it was improved with increases in the Ag concentration. This is probably due to the addition of Ag nanoparticles increasing the polymer suspension concentration, which might have resulted in the formation of thicker nanofibers, the same trend as the results of Maleki, et al. [60,61].

**Figure 3:**
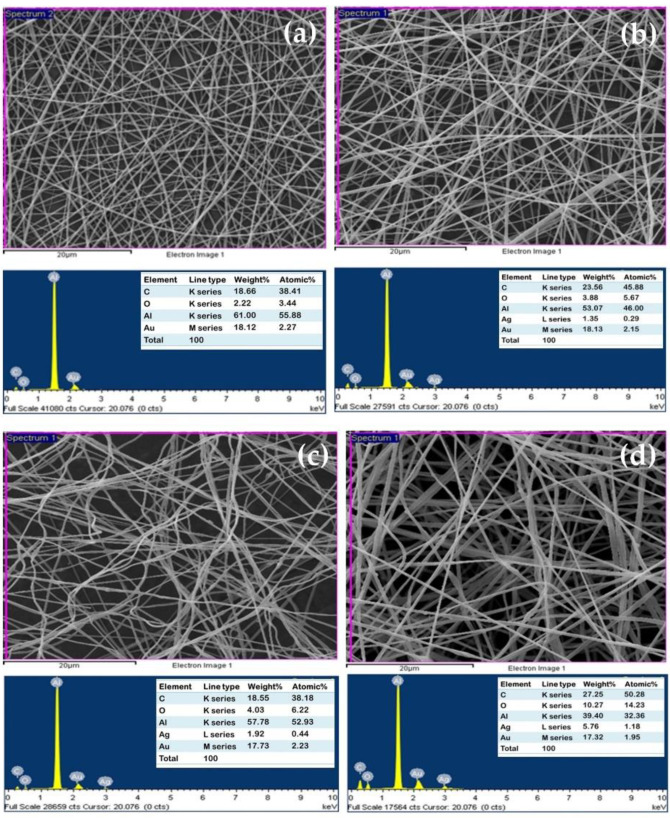
SEM images and EDS spectra of electrospun fiber mats based on PLA: (**a**) neat PLA, (**b**) PLA/Ag-1, (**c**) PLA/Ag-3, and (**d**) PLA/Ag-5, respectively.

In addition, to confirm the element compositions, the energy dispersive X-ray spectrum of all fabricated nonwoven fiber mats based on PLA was investigated. The results revealed that the nonwoven fiber mats of PLA composited with Ag at different concentrations displayed significant peaks of Ag, which reflect the composition of Ag elements in the nonwoven fiber mats. However, it did not display this peak in the EDS spectra of neat PLA, as shown in Figure 3. This result suggested that the silver was successfully incorporated in the PLA nanofibers. The amounts of silver within the PLA nanofibers were approximately 1.35%, 1.92%, and 5.76% for PLA/Ag-1, PLA/Ag-3, and PLA/Ag-5, respectively. The amounts of Ag content within nonwoven fiber mats increased with an increase of AgNO_3_ concentrations during the prepared PLA suspensions for the electrospinning process. Furthermore, all nonwoven fiber mats show the elemental characteristic peaks of the PLA molecule, i.e., carbon (C) and oxygen (O). Strong signal peaks of Al and Au came from the substrate of the electrospun fiber collector and coating gold prior to SEM investigation, respectively.

### 3.2. Fourier Transform Infrared Spectroscopy

**Figure 4:**
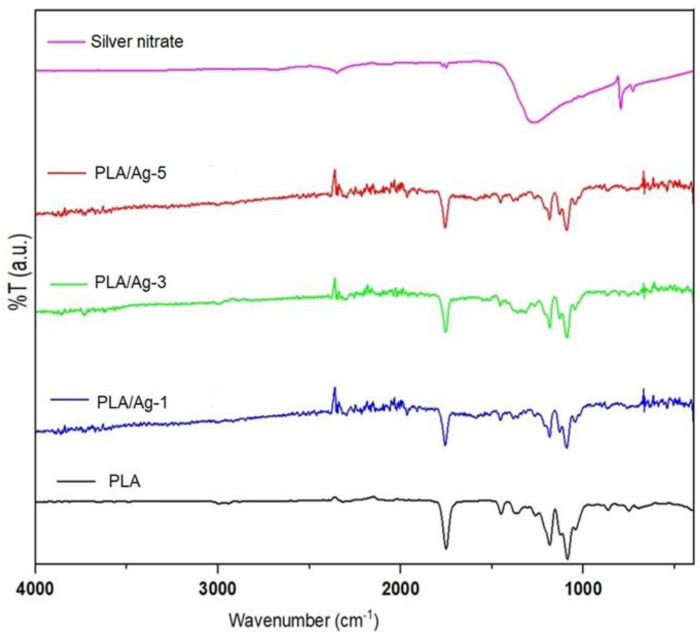
FTIR spectra of silver nitrates, PLA, and PLA/Ag electrospun fiber mats.

The incorporation of silver into PLA nanofibers, as well as the chemical reaction between those molecules upon electrospinning, were confirmed by FTIR, and the results are reported in Figure 4. All the IR spectra of nonwoven fiber mats exhibited peaks at approximately 1184 cm^−1^ and 1755 cm^−1^, which can be indexed to the characteristic of C=O stretching and C-O stretching of the carboxyl group in the PLA molecules [62,63]. In addition, the small and weak peaks around 2995 and 3350 cm^−1^ are associated with the stretching of CH_2_ and O-H, respectively [64]. Silver nitrate used as a precursor to produce Ag nanoparticles was also investigated for comparison. In the case of the nanocomposite nanofiber mats, a strong peak around 1190 cm^−1^ was displayed, and the position of this peak changed little, while its intensity gradually weakened and the intensity and peak shifted to lower wavenumbers. This indicates that the addition of silver nitrate has an intermolecular reaction with PLA molecules. Furthermore, a broad band in the range of 600 to 800 cm^−1^ could be attributed to Ag–O vibration [65]

### 3.3. Differential Scanning Calorimetry (DSC) Analysis

**Figure 5:**
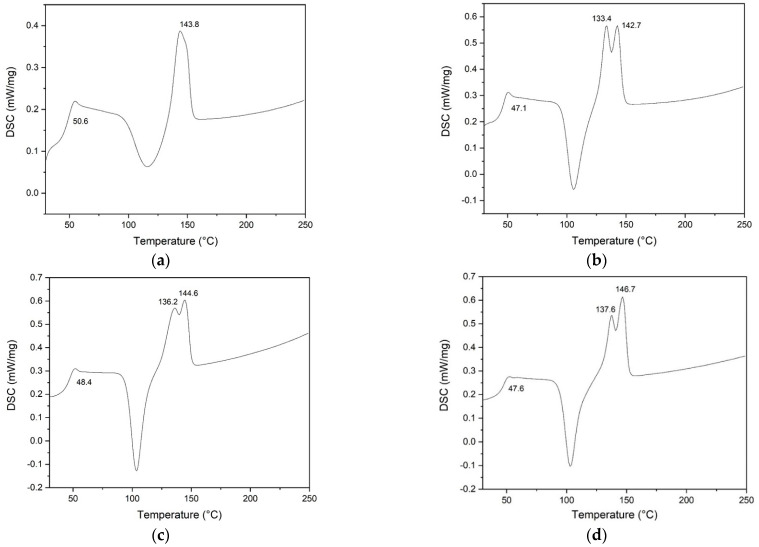
DSC thermograms for the electrospun PLA fibers: (**a**) PLA, (**b**) PLA/Ag-1, (**c**) PLA/Ag-3, and (**d**) PLA/Ag-5, respectively.

DSC thermogram results are presented in Figure 5. The values of glass temperature (*T*_g_), melting temperature (*T*_m_), melting enthalpy $\left(∆H_{m}\right)$, and the degree of crystallinity of the electrospun PLA fibers containing the various Ag concentrations were recorded in Table 1. Observably, the fabricated nonwoven fiber mats from PLA composited with Ag dominantly display two peaks for the melting temperature, corresponding to it not being homogeneity miscible between Ag particles and PLA matrices, which is in agreement with the research results of Domingues et al. [66]. Additionally, we found that the contents of Ag nanoparticles within the PLA matrix slightly affected the thermal properties of nonwoven fiber mats in both *T*_g_ and *T*_m_ values, as shown in Table 1. In addition, as expected, the degree of molecular order of the chains is higher in PLA composite nanofibers than in neat PLA nanofiber, as shown by the enthalpy values of 35.75, 33.58, 27.99, and 25.42 J/g for PLA/Ag-1, PLA/Ag-3, and PLA/Ag-5, respectively. This result suggests crystallization of the polymer, resulting in increases in the degree of crystallinity of PLA nonwoven fiber mats, as illustrated in Table 1. This result shows that the addition of silver particles acts as a nucleating agent, which increases the melting temperature and degree of crystallinity of PLA. This means that the silver particles influence intermolecular interactions or chain flexibility of PLA polymer chains. This result agrees with the results reported by Greco et al. [67] and Oksiuta and coworkers [68].

### 3.4. Thermal Gravimetric Analysis (TGA)

**Figure 6:**
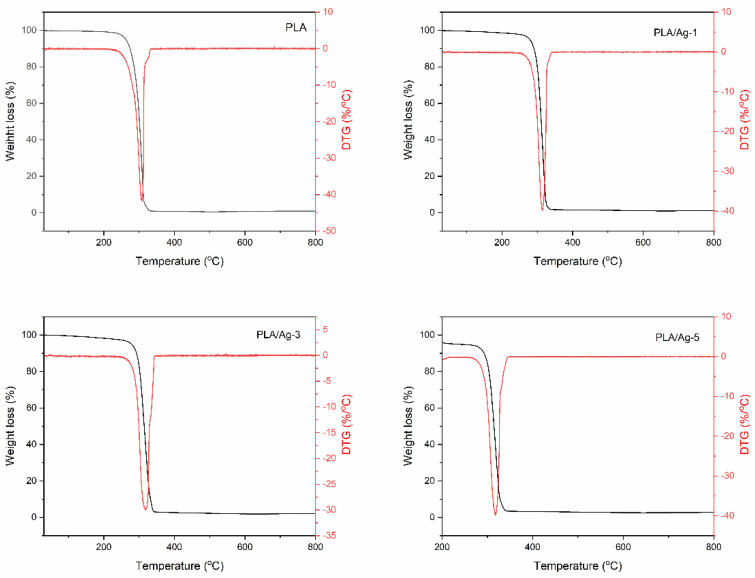
TGA and DTG analysis for electrospun nanofiber mats from neat PLA and PLA embedded with different Ag concentrations.

TGA was carried out to investigate the thermal stability of the electrospun fiber mats from neat PLA and the effect of silver loading on the thermal stability of the PLA. Figure 6 shows the TGA and DTG curves, respectively, of the electrospun fiber mats of pure PLA and the PLA/Ag nanocomposite nanofibers with different Ag contents. Following Figure 6, as can be seen, three degradation steps are observed for each of the electrospun fiber mats. The first stage occurred below 100 °C was attributed to evaporating the adsorbed moisture on the surface of electrospun fiber mats. The second stage, between 135 °C and 260 °C, was due to the primary degradation of PLA molecules, and the last stage, which resulted in the decomposition of PLA main chains, was from 260 °C to 345 °C. In this stage, the main maximum on the DTG curve, which shows the highest rate of thermodestruction, was recorded at the temperature of 308 °C. In the case of PLA/Ag nanocomposite fibers, it seems no differences were observed when comparing PLA/Ag-1, PLA/Ag-3, and PLA/Ag-5 thermograms. The temperature of the third degradation phase increased with increases in the Ag concentrations. Additionally, the maximum on the DTG analysis curves was also recorded at temperatures of 314, 317, and 318 °C for PLA/Ag-1, PLA/Ag-3, and PLA/Ag-5, respectively. That is, the introduction of Ag nanoparticles in the PLA matrix increases the heat resistance of PLA-Ag composites. From the result, the thermal stability of the neat PLA nanofiber mat seemed lower as compared to the nanocomposite nanofiber mats. It is suggested that the introduction of Ag nanoparticles in the PLA matrix can improve the thermal stability of the PLA electrospun fiber mats. This behavior is possibly due to the reduction in the mobility of the molecular chain of PLA matrices in the electrospun fiber mats. It is well known that with the reduced chain mobility, the interaction of chain transfer will be suppressed, and consequently the degradation process will be slowed, and decomposition will take place at higher temperatures [69,70]. This result is in good agreement and similar to the results of Mbhele et al. [71] and He et al. [72].

### 3.5. Water Absorbency

**Figure 7:**
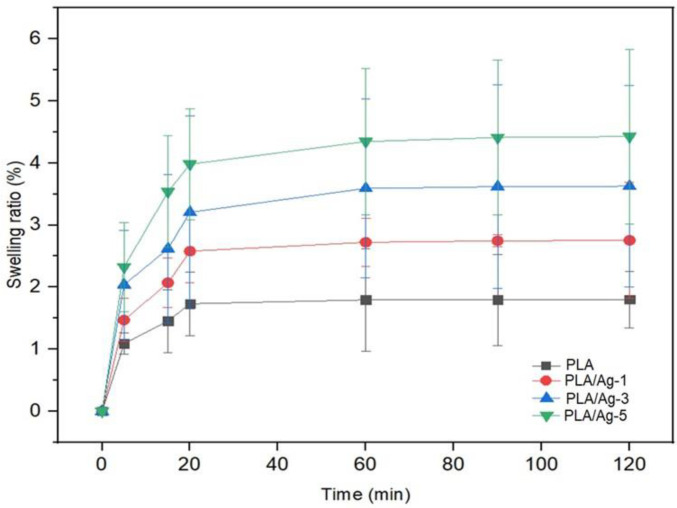
Swelling ratio of nanofibers with various concentrations as a function of time.

In this investigation, the swelling ratios of nanofibers of PLA and PLA composited with Ag nanoparticles at different concentrations are shown in Figure 7. From this study, we found the nonwoven fiber mats exhibit an equilibrium absorbency of water in the range of 1–4%, depending on their compositions. These structured fiber mats did not absorb water very much due to the effect on the functionality of the chemical structure of PLA molecules; therefore, they show highly hydrophobic properties. In Figure 6, all nonwoven fiber mats exhibited a high initial water adsorption rate and then reached equilibrium within 20 min. At the given contact time, the nonwoven composite PLA/Ag fiber mats showed higher values of swelling ratio than neat PLA. This is probably due to the miscibility between the silver particles and PLA matrices. The swelling ratio of nonwoven fiber mats increased when increasing the concentration of Ag nanoparticles, which resulted in more space or free volume on the interface of composites; hence, it illustrates a high swelling ratio when comparing to the neat PLA nanofibers.

### 3.6. Antibacterial Testing

**Figure 8:**
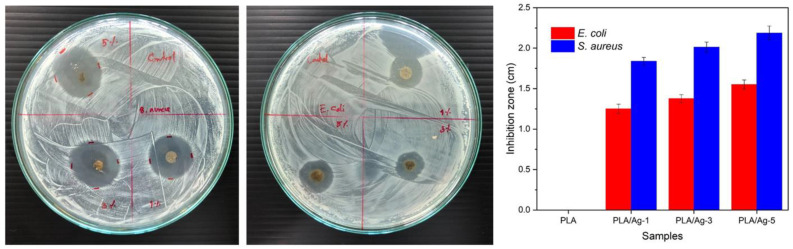
Diffusion test for antibacterial activity of neat PLA, PLA/Ag-1, PLA/Ag-3, and PLA/Ag-5 against *S. aureus* and *E. coli* bacteria strains.

Antibacterial activities of nonwoven fiber mats fabricated from neat PLA and PLA composited Ag nanoparticles at various concentrations were evaluated against both Gram-positive (*S. aureus*) and Gram-negative (*E. coli*) bacteria by using disk diffusion susceptibility and plate colonies count methods. Our results demonstrated that no antibacterial activity was detected for neat PLA electrospun fiber mats, which were used as control materials, as illustrated in Figure 8. In contrast, the antibacterial efficacy of both *S. aureus* and *E. coli* of the PLA composite nanofiber mats gradually increased with an increase in the amounts of Ag nanoparticles, as can be seen in Figure 8. In addition, the size of inhibition zones also depends on the type of bacterial strain. In this study, all the obtained nonwoven fiber mats against *S. aureus* bacteria were higher than *E. coli* bacteria. This is probably due to the difference in the bacterial structure characteristics, resulting in the difference in the sensitivity and efficacy of antibacterial activity, as well as is in agreement with those of the available scientific literature [73,74,75].

**Figure 9:**
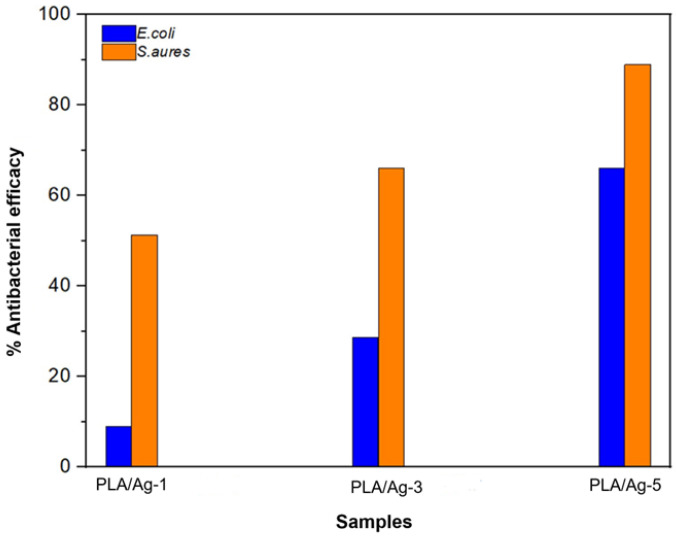
Quantitative antibacterial activity of fabricated nonwoven fiber mats on *E. coli* (blue) and *S. aureus* (orange).

More precisely, the quantitative analysis of electrospun fiber mats’ antibacterial activities is demonstrated in Figure 9. The results show that the PLA/Ag exhibits a significant increase in the antibacterial properties with a larger amount of Ag nanoparticle content. Bacteria and culture media with neat PLA nanofiber mats were used as a control. The number of colonies was counted after sampling the bacteria found in different samples, as depicted in Table 2. PLA/Ag-5 nanofiber mats exhibited the highest antibacterial efficacies, both in *S. aureus* and *E. coli*, among three of the obtained composite nonwoven fiber mats.

**Figure 10:**
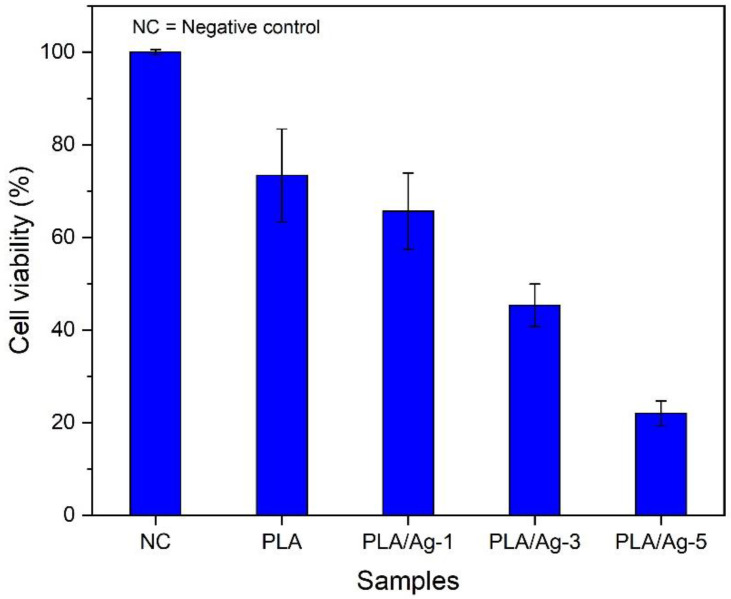
Percentage of cell viability of L929 mouse fibroblast cells by MTT assay of different mats over 24 h of culture. NC: Cells and culture media without any samples. The experiments were repeated in triplicate.

One of the essential characteristics of wound dressing is the growth and differentiation of cells in the wound site. Thus, to determine the cytotoxicity of the obtained nonwoven fiber mats to fibroblasts, MTT assays were carried out to investigate cell viability. The MTT assay is a standard for testing that has been reported [76]. The results showed that all fabricated nonwoven fiber mats have affected cell viability. In particular, the PLA/Ag electrospun fiber mats exhibited a significant influence on the amount of surviving cells. In addition, as expected, the cell viability decreased with increases in the Ag nanoparticles within PLA matrices, as represented in Figure 10. The percentage of viable cells showed approximately 73.41 ± 10.02, 63.69 ± 8.20, 35.36 ± 4.61, and 21.98 ± 2.70 for nonwoven fiber mats prepared from PLA, PLA/Ag-1, PLA/Ag-3, and PLA/Ag-5, respectively. Following this line of reasoning, we tentatively suggest that our results indicate that Ag nanoparticles have much effect on the survival of the cells. This study concurred with the findings of research that showed Ag nanoparticle concentrations have a direct inhibitory effect on cell growth.

## 4. Conclusions

In this study, we successfully designed and prepared antibacterial nonwoven fiber mats of PLA and PLA hybridized with Ag nanoparticles, smoothly and uniformly without any beads, via an electrospinning technique. The results showed that the average diameters of the PLA nanofibers containing the Ag nanoparticles were larger than those without those particles. Also, this diameter size is proportional to the amount of Ag nanoparticle contents. Additionally, the contents of Ag nanoparticles incorporated within the PLA matrix show insignificant effects on the thermal properties of the nonwoven fiber mats. The crystallinity of the nonwoven fiber mats from PLA composited with Ag nanoparticles was a bit higher than those of fiber mats of the neat PLA. FTIR and EDS spectra indicated that Ag nanoparticles were merely incorporated in the PLA matrices without any chemical bonding between the particles of silver and the PLA chains. Accordingly, the antibacterial activity results revealed a more substantial growth inhibitory effect in the short term of the PLA/Ag-5, concerning the Ag nanoparticles concentration, against both *S. aureus* and *E. coli*, with a more pronounced action towards the Gram-positive bacteria than Gram-negative bacteria. In addition, all produced nonwoven fiber mats induced a reduction in cell viability for the L929 fibroblast cell line in the cell toxicity study. Overall, we suggest that the nonwoven fiber mats made here showed excellent morphological and thermal properties as well as antibacterial efficacy; moreover, the possibility of modifying functionalities of the materials can be adjusted with the ratio between PLA and Ag nanoparticles for potential use in biomedical applications.

## Figures and Tables

**Table 1:** Summarized results of DSC analysis of PLA/Ag electrospun fibers mat.

Sample Codes	*T*_c_ (°C)	*T*_m_ (°C)	${∆H}_{m}$ (J/g)	$\chi_{c}\;(\%)$
PLA	50.6	143.8	25.42	27.33
PLA/Ag-1	47.1	133.4, 142.7	27.99	30.40
PLA/Ag-3	48.4	136.2, 144.6	33.58	37.22
PLA/Ag-5	47.6	137.6, 146.7	35.75	40.46

**Table 2:** Antibacterial efficacies of PLA/Ag electrospun fibers mat.

Samples	*E. coli*		*S. aureus*	
	Number of Living Bacteria (CFU/mL)	Reduction of Living Bacteria (%)	Number of Living Bacteria (CFU/mL)	Reduction of Living Bacteria (%)
PLA	1.8 × 10^4^	0.0	8.4 × 10^4^	0.0
PLA/Ag-1	1.7 × 10^4^	9.0	4.1 × 10^4^	51.3
PLA/Ag-3	1.3 × 10^4^	28.6	2.8 × 10^4^	66.0
PLA/Ag-5	6.4 × 10^3^	66.0	9.3 × 10^3^	89.0

## Data Availability

Data are contained within the article.
